# Application of FTA technology for sampling, recovery and molecular characterization of viral pathogens and virus-derived transgenes from plant tissues

**DOI:** 10.1186/1743-422X-2-45

**Published:** 2005-05-18

**Authors:** Joseph Ndunguru, Nigel J Taylor, Jitender Yadav, Haytham Aly, James P Legg, Terry Aveling, Graham Thompson, Claude M Fauquet

**Affiliations:** 1 International Laboratory of Tropical Agriculture Biotechnology, Donald Danforth Plant Science Center, 975 North Warson Road, St. Louis, MO 63132, USA; 2 Ministry of Agriculture and Food Security, Plant Protection Services, Box 1484, Mwanza, Tanzania; 3 Department of Genetics, Faculty of Agriculture, Cairo University, Giza, Egypt; 4 International Institute of Tropical Agriculture-Eastern and Southern Regional Center and Natural Resource Institute, Box 7878, Kampala, Uganda; 5 ARC-Roodeplaat Vegetable and Ornamental Plant Institute, Private Bag X293, Pretoria 0001, Pretoria, South Africa

## Abstract

**Background:**

Plant viral diseases present major constraints to crop production. Effective sampling of the viruses infecting plants is required to facilitate their molecular study and is essential for the development of crop protection and improvement programs. Retaining integrity of viral pathogens within sampled plant tissues is often a limiting factor in this process, most especially when sample sizes are large and when operating in developing counties and regions remote from laboratory facilities. FTA is a paper-based system designed to fix and store nucleic acids directly from fresh tissues pressed into the treated paper. We report here the use of FTA as an effective technology for sampling and retrieval of DNA and RNA viruses from plant tissues and their subsequent molecular analysis.

**Results:**

DNA and RNA viruses were successfully recovered from leaf tissues of maize, cassava, tomato and tobacco pressed into FTA^® ^Classic Cards. Viral nucleic acids eluted from FTA cards were found to be suitable for diagnostic molecular analysis by PCR-based techniques and restriction analysis, and for cloning and nucleotide sequencing in a manner equivalent to that offered by tradition isolation methods. Efficacy of the technology was demonstrated both from sampled greenhouse-grown plants and from leaf presses taken from crop plants growing in farmer's fields in East Africa. In addition, FTA technology was shown to be suitable for recovery of viral-derived transgene sequences integrated into the plant genome.

**Conclusion:**

Results demonstrate that FTA is a practical, economical and sensitive method for sampling, storage and retrieval of viral pathogens and plant genomic sequences, when working under controlled conditions and in the field. Application of this technology has the potential to significantly increase ability to bring modern analytical techniques to bear on the viral pathogens infecting crop plants.

## Background

The viral pathogens that infect crop plants constrain food production and economic development throughout the world's agricultural regions. Viral diseases are difficult to prevent, and once established few means are available to counter their impact on yield. As a result, development and deployment of resistance crop varieties remains the most effective manner in which to combat the evolving threats presented by plant viral diseases. Underpinning such efforts is the need for robust diagnostic capacities to identify the species and strains of viral pathogens infecting crop plants and their related wild species, and to understand their distribution within a given geographical region.

Access to simple, low cost tools for the molecular study of plant viral pathogens is central to generating the knowledge and improved germplasm required by scientists, breeders and farmers to combat these diseases and maximize crop yields. Effective methods for sampling, storage and retrieval of viral pathogens from infected plant tissues allows not only identification of the viral pathogens but also detailed molecular study of their genomes, generating increased understanding of their epidemiology, etiology and evolution. Diagnostic technologies are also required for virus indexing to facilitate certification of pathogen-free materials for the collection, maintenance and international exchange of the elite germplasm on which required plant improvement programs are based.

Molecular characterization of the viruses that infect plant material is currently achieved by direct electrophoretical isolation from total nucleic acid, followed by cloning and subsequent analysis, or amplification of full or partial genomic sequences by polymerase chain reaction (PCR). PCR is the more powerful technique due to its ability to recover viral sequences and whole genome components from very low viral titres, and is now the preferred approach for most applications. Currently, total viral and genomic nucleic acids are isolated from infected tissues by methods such as Dellaporta et al. [1] which involve multi-step protocols for DNA or RNA extraction, precipitation and purification. A frequent limitation for studying viruses at the molecular level is the ability to reliably obtain high quality nucleic acids from putatively infected plant material. Plant tissues to be analyzed must be collected and preserved in order to maintain integrity of the nucleic acids until they can be processed. This poses challenges when sample numbers are large and when working in the field, most especially in the tropical and sub-tropical regions where plant viral pathogens are abundant. Field studies are thus constrained by the resources required for sample preservation and transportation, placing restrictions on the number of samples that can be collected in a given time and the size and remoteness of the regions that can be effectively surveyed. Timely processing and/or storage of the samples before they spoil can also be problematic in locations where access to well equipped laboratory facilities is limited.

We report here the use of FTA technology for efficient sampling and recovery of viral pathogens from infected leaf tissues and their subsequent molecular analysis. Utilising the geminiviruses that infect maize (*Zea mays*), cassava (*Manihot esculenta*) and tomato (*Lycopersicum esuclentum*), in addition to Tobacco mosaic virus (TMV), Potato virus Y (PVY) and Tobacco etch virus (TEV), we provide evidence that diagnostic techniques can be applied to both DNA and RNA viruses eluted from FTA cards in a manner equivalent to conventional isolation methods, and that this cost-effective technology significantly simplifies the sampling and analysis of diseased plants in both the laboratory and field environments.

FTA is a paper-based technology designed for the collection and archiving of nucleic acids, either in their purified form or within pressed samples of fresh tissue. Proprietary chemicals impregnated into the paper act to lyze cellular material and fix and preserve DNA and RNA within the fibre matrix [2]. After a short drying period, pressed samples can be stored at room temperature for extended periods and processed when required. Nucleic acids are recovered by removing small punches from the pressed area and washing with simple reagents. RNA and smaller DNA molecules, such as plasmids and viral genomic components, are eluted by a simple extraction buffer and used as template for amplification by PCR. Genomic DNA remains attached to the paper matrix but is available for amplification by PCR when the paper punch is included in the PCR reaction mix. Advantages of FTA technology have been realized for human DNA processing and forensic applications [3], for wildlife DNA samples [4] and applied to PCR-based genotyping [5,6] but have not been well documented for use with plant pathogens. Recognizing the potential benefits this technology could bring to sampling and molecular study of viral crop diseases, we tested the efficacy of FTA for retrieval of viral pathogens from infected leaf tissues and for the detection of viral-derived transgene sequences in transgenic plants.

## Results

### Use of FTA for sampling, retrieval and PCR-based analysis of DNA viruses

Replicated samples from newly unfolded, symptomatic leaves of cassava, maize, tomato and *Nicoticana benthamiana *plants infected with geminiviruses were used to study the efficacy of FTA technology for sampling, retrieval and molecular analysis of these viruses. Geminiviruses are composed of monopartite or bipartite genomes of ssDNA, 2.7–2.8 kb in size. As such they can be eluted from the FTA paper matrix after appropriate washing steps and used as template for PCR diagnostic analysis. In order to compare efficacy of FTA technology compared to traditional methods, tissue from each sample was split in two, with one pressed onto an FTA card and the other used for DNA extraction via the Dellaporta method [1].

**Figure 2:**
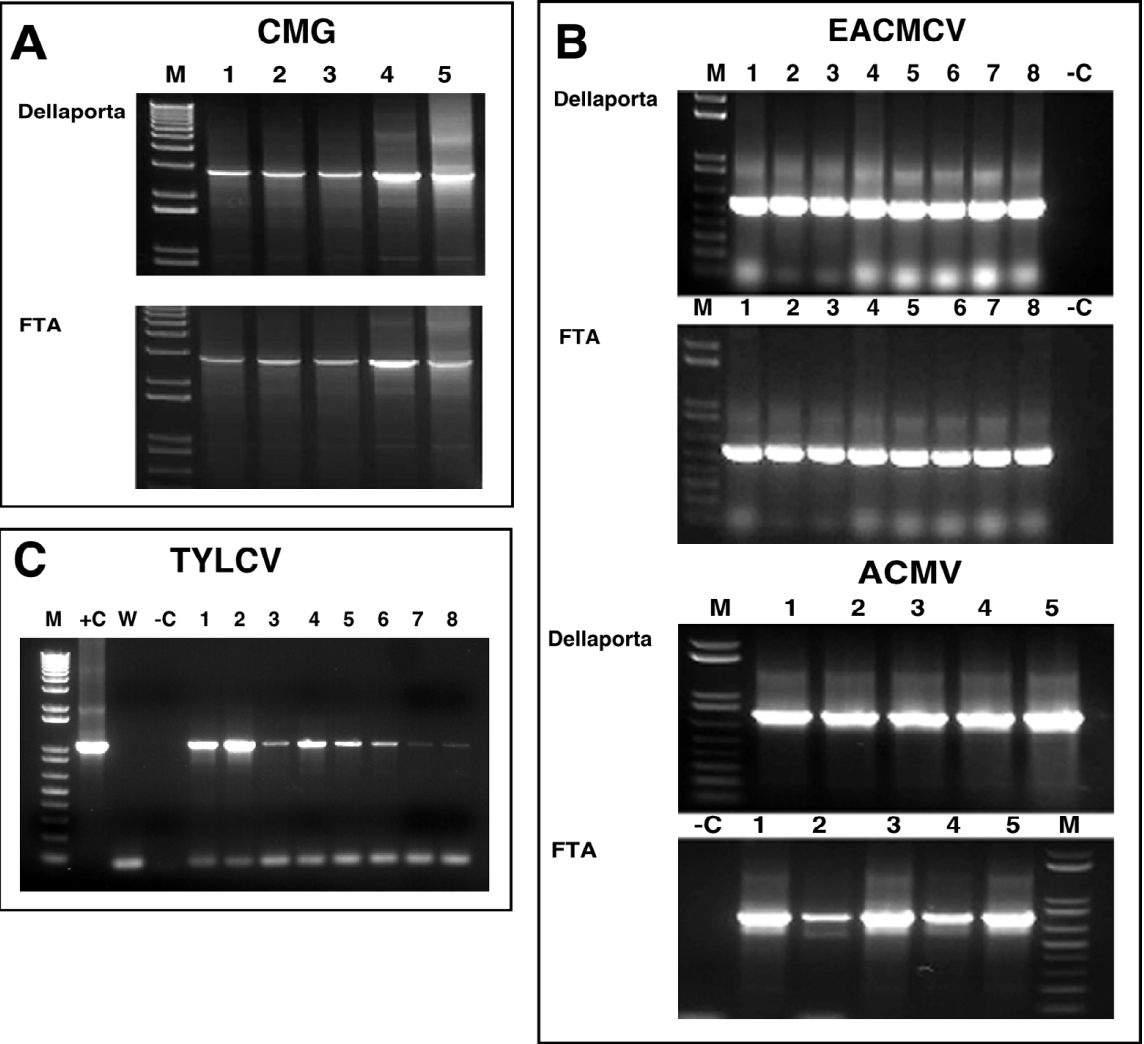
**PCR amplification of geminiviruses components from symptomatic leaves of greenhouse-grown plants using traditional DNA isolation methods and FTA technology**. (a) amplification of the 2.8 kb B component of cassava mosaic geminiviruses using universal primers UniF and UniR (Table 1) from independent infected plants. Template DNA was obtained either by extraction and purification of total DNA according to Dellaporta et al. [1] (0.2 μg template) or by elution of viral DNA from leaf tissue pressed onto FTA Classic Cards. (b) amplification of East African cassava Cameroon virus (EACMCV) (lanes 1–8) and African cassava mosaic virus (ACMV) (lanes 1–5) from diseased cassava plants isolated by Dellaporta-based methods (0.2 μg template) or from viral DNA isolated from leaf tissue pressed onto FTA cards. A 550 bp fragment of the B genomic component of EACMCV was amplified using primers EAB555/F and EAB555/R (Table 1) and a 500 bp fragment of the coat protein gene from the A genomic component of ACMV generated using primers JSP001 and JSP002 (Table 1). (c) amplification of the 1.07 kb C1 gene of the Egyptian strain of the monopartite tomato yellow leaf curl virus eluted from infected tomato (lanes 1,2,5 and 6) and *N. benthamiana *(lanes 3, 4, 7 and 8) leaves pressed onto FTA cards. Increasing the time which paper punches were soaked in elution buffer from 30 minutes (lanes 1–4) to 12 hours (lanes 5–8) increased the signal strength of the amplified viral sequence in both plant species. In all cases, M: marker, +C: positive control, -C: negative control, W: water control. CMG – cassava mosaic geminiviruses; ACMV – African cassava mosaic virus; EACMV – East African cassava mosaic virus; TYLCV – Tomato yellow leaf curl virus.

Universal primers UniF and UniR, designed to amplify the near full-length cassava geminivirus DNA-A component (Table 1), were used to detect the presence of cassava mosaic geminiviruses (CMG) in infected cassava tissues. All plants sampled in this manner generated signals of the appropriate size. Signals were similar whether the DNA was eluted from FTA cards or extracted by the Dellaporta method (Fig. 2a), demonstrating that PCR amplification of sequences equivalent to the whole genomic component of a CMG was possible from samples preserved on FTA cards, in a manner equal to that from traditional DNA isolation techniques.

**Table 1:** Oligonucleotides used for PCR amplification of viral and transgene sequences

**Primer**	**Sequence **(5'-3')	**Target sequence**
EAB555/F	(5'-TACATCGGCCTTTGAGTCGCATGG-3')	EACMV DNA B
EAB555/R	(5'-CTTATTAACGCCTATATAAACACC-3')	EACMV DNA B
JSP001	(5'-ATGTCGAAGCGACCAGGAGAT-3')	ACMV (AV1/CP)
JSP002	(5'-TGTTTATTAATTGCCAATACT-3')	ACMV (AV1/CP)
UniF	(5'-KSGGGTCGACGTCATCAATGACGTTRTAC-3')	CMGs DNA A
UniR	(5'-AARGAATTCATKGGGGCCCARARRGACTGGC-3')	CMGs DNA A
MSVF	(5'-ATCCCTCCAAATTCCGACAC-3')	MSV
MSVR	(5'-TCCATGTACAAAGCTCCTCT-3')	MSV
C1F	(5'-GCAGATCTATGCCTCGTTTATTTAAAATATATGC-3')	TYLCV
C1R	(5'-GCGGTACCTTACGCCTTATTGGTTTCTTCTTGGC-3')	TYLCV
TMVF	(5'-GCGGTGGCGGCCGATCCATGGAACTTACAG-3')	TMV
TMVR	(5'-GATTCGAACCCCTCGCTTTAT-3')	TMV
POT1	(5'-gacgaattcTGYGAYGCBGATGGYTC-3')	TEV & PVY
POT2	(3'-ACCACRTADCTBTTAcctaggtcag-5')	TEV & PVY
AC1F	(5'-ATGAGAACTCCTCGTTTTAGAA-3')	ACMV-Kenya AC1
AC1R	(5'-ATGAGAACTCCT CGTTTTAGAA-3')	ACMV-Kenya AC1
MP141	(5'-ATGATTGAACAAGATGGATTGCAC-3')	*Npt*II coding sequence
MP142	(5'-TCAGAAGAACTCGTCAAGAAGGCG-3')	*Npt*II coding sequence

EACMV – East African cassava mosaic virus; ACMV – African cassava mosaic virus; MSV – maize streak virus; TYLCV – tomato yellow leaf curl virus; TMV – tobacco mosaic virus; TEV – tobacco etch virus; PVY – potato virus Y

*EAB555/F *&*EAB555/R: *PCR conditions consisted of 30 cycles of 94°C for 1 min, 58°C for 1 min. and 72°C for 2 mins.

*JSP001 *&*JSP002*: PCR conditions consisted of 30 cycles of 94°C for 1 min, 45°C for 1 min. and 72°C for 2 mins.

*Uni F *&*Uni R*: K = G + T, R = A + G, S = G + C. PCR conditions consisted of 30 cycles of 94°C for 1 min., 58°C for 1 min. and 72°C for 2 min.

*MSV F *&*MSVR *: PCR conditions consisted of 30 cycles of 94°C for 1 min, 59°C for 1 min and 72°C for 2 mins.

*C1F *&*C2R*: PCR conditions consisted of 94°C for 10 mins followed by 35 cycles of 94°C for 30 secs., 60°C for 1 min and 72°C for 1.5 mins followed and extension of 7 mins at 72°C.

*AC1F *&*AC1R *: PCR conditions consisted 94°C of 5 mins followed by 35 cycles of 94°C for 30 secs, 58°C for 1 min. and 72°C for 1 mins followed by extension of 10 mins at 72°C.

*MP141 *&*MP142*: PCR conditions consisted 5 mins. at 94°C followed by 35 cycles of 94°C for 30 secs, 58°C for 1 min. and 72°C for 1 mins. followed by extension time of 10 mins. at 72°C.

For FTA technology to be effectively used as a routine tool for PCR-based geminivirus diagnostics, it must allow for differentiation of the viral species infecting a given plant. Greenhouse-grown cassava plants infected with East African cassava mosaic Cameroon virus (EACMCV) or African cassava mosaic virus (ACMV) were tested for the presence of the specific geminivirus species by pressing symptomatic leaves onto FTA cards and by isolation of total DNA by the Dellaporta method. Two primer pairs, EAB 555F/EAB555R and JSP 001/ JSP 002 [7], designed to amplify all strains of EACMV-like and ACMV-like geminiviruses respectively (Table 1), were employed to test for presence of the viral pathogens. Both ACMV and EACMV were detected from samples collected on FTA cards. PCR product characteristics were similar between the paper-based and traditional protocols in all thirteen plants analyzed in this manner (Fig. 2b). In some FTA derived samples, signal strength from the amplified products was lower than that generated from 0.2 μg of DNA used as template from the Dellaporta method but in all cases remained easily detectable.

FTA technology was also used to sample tomato and *Nicotiana bethamiana *plants infected with an Egyptian strain of the monopartite geminivirus, Tomato yellow leaf curl virus (TYLCV) (GenBank:AY594174). Template DNA eluted from symptomatic leaves pressed onto FTA cards yielded expected bands in all plants tested with primer pair C1F and C1R designed to amplify the *C1*, replication associated gene (1.074 kb) from this virus (Fig. 2c).

**Figure 3:**
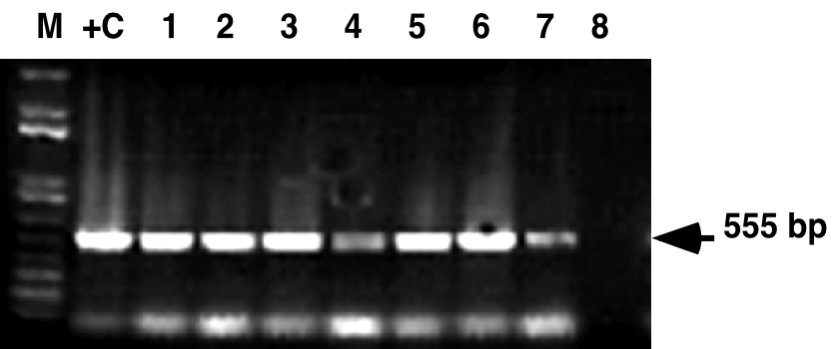
**PCR amplification from serial dilutions of viral DNA elution from FTA cards**. Serial dilutions of plasmid DNA carrying known amounts of the B genomic component of East African cassava Cameroon virus (EACMCV) were mixed with leaf sap extract from healthy cassava plants and spotted onto FTA cards. Viral DNA was eluted from FTA cards and used as template for PCR amplification of a 550 bp fragment using primers EAB555F and EAB555R guided experimental design (Table 1). 0.2 μg DNA was used as template in the positive control lane (C+).

Investigations were carried out to further quantify the ability of FTA technology to fix, store and release geminivirus genomic components. Recombinant plasmid DNA carrying the B component of EACMCV [8] in quantities as follows: 0.8, 0.4, 0.2, 0.16, 0.08, 0.05, 0.04, and 0.001 μg, were mixed with 8 μl of sap extracted with distilled water from healthy cassava leaves. When these elutions were used for PCR, amplification using primers EAB 555F and EAB555R was successful in all cases except the lowest, where only 0.001 μg was loaded onto the card (Fig. 3). In our hands, therefore, FTA technology can be reliably employed to detect geminivirus loads within infected leaf tissues of cassava above the 40 picogram level.

### Improvements to existing protocols

Two important improvements were developed during the above studies and incorporated into the protocol supplied by Whatman International in order to increase the quality and yield of eluted virus from FTA cards. It was found that in some cases, paper punches removed from leaf tissues pressed into FTA cards retained green pigment after washes with TE buffer and FTA purification reagent. Release of these pigments during the final elution step inhibited subsequent PCR amplification of viral sequences. Addition of one or (if required) two five-minute washes with 70% ethanol prior to the FTA purification reagent step resulted in removal of most green pigmentation from the punches and ensured that the final elution was free from contaminates. It was also found that the yield of eluted viral DNA could be increased, and significantly enhanced amplification of the target sequence achieved, if the processed paper punches were soaked overnight in elution buffer at 4°C, compared to the 15–20 minutes at room temperature recommended by the manufacturer (Fig. 2c). These additional steps have become standard procedures in our laboratory.

### Cloning, sequencing and restriction analysis of viral elutes from FTA

Nucleic acid sequencing provides the highest level of viral diagnostic analysis available and facilitates development of additional tools for subsequent molecular-based studies of these pathogens. To determine whether viral DNA stored on FTA cards was suitable for downstream, high fidelity characterization and analysis, a 550 bp fragment from the DNA B component of EACMCV was PCR-amplified from greenhouse grown, CMG infected plants using primers EAB555/F and EAB55/R (Table 1). Template DNA eluted from FTA cards and from conventional extraction methods were directly compared. PCR products were purified and cloned into the pGEM-T Easy vector. Two clones from each DNA recovery process were sequenced in both orientations and the DNA nucleotide sequences compared by multiple alignment using MegaAlign software of the DNASTAR computer package. No significant nucleotide sequence variation was observed when a corresponding EACMCV DNA-B sequence fragment (GenBank:AF112355) was compared to the clones sequenced in this study (results not shown). Clones from FTA-processed DNA were comparable with those from phenol extracted DNA, with nucleotide sequence comparison showing 99.8% identity between clones derived from FTA technology and the traditional method of viral DNA processing and phenol purification. These results indicate that viral DNA within plant tissues fixed on FTA card retains fidelity of its nucleotide sequences throughout the sampling, storage and recovery processes. When combined with the ability described above to clone and amplify whole genome sized sequences, we are confident that FTA technology can be employed to generate full genomic sequence data for geminiviruses and to produce infectious clones of these pathogens isolated from diseased plant tissues.

### Use of FTA to sample, recover and diagnose viruses from field-grown crop plants

**Figure 1:**
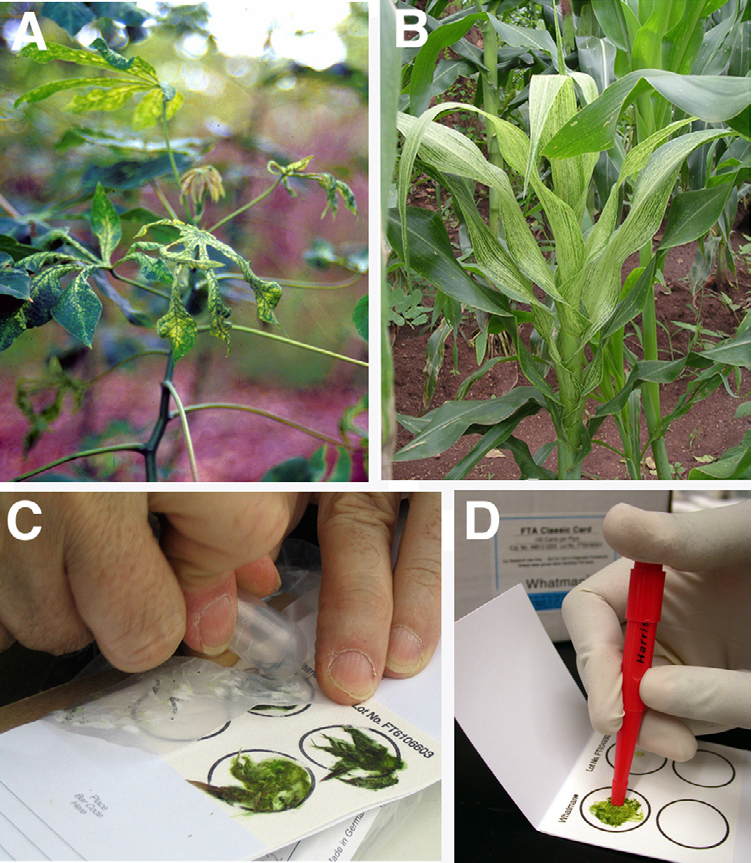
**Disease symptoms on field grown cassava and maize plants and FTA sampling method**. (a) cassava plant in Western Kenya showing severe cassava mosaic disease symptoms (b) severe maize streak virus symptoms on maize plant in farmer's field in Malawi (c) symptomatic leaves are pressed into FTA Classic Cards (d) 2 mm diameter punches being removed from FTA Classic Card for subsequent viral DNA elution and molecular analysis

**Figure 4:**
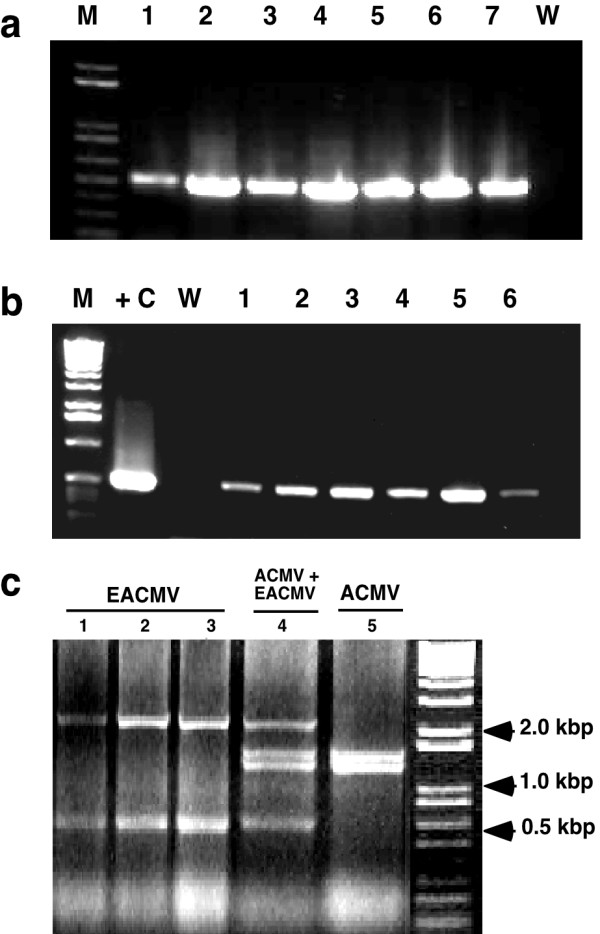
**Analysis of geminivirus DNA eluted from FTA-preserved leaf tissues of infected maize and cassava plants growing in farmer's fields in Kenya and Malawi**. (a) detection of maize streak virus from infected plants in Malawi. Primers MSV-F and MSV-R (Table 1) were used to amplify a 500 bp fragment from the conserved region of this monopartite geminivirus. (b) detection of East African cassava mosaic virus-like sequences from leaf tissues pressed onto FTA cards plants in Malawi. Primers EAB555F and EAB555R (Table 1) were used to amplify a 550 bp fragment of the B genomic component. (c) Restriction analysis of whole A genomic components (2.8 kb) of East African cassava mosaic virus (EACMV) and African cassava mosaic virus (ACMV) isolated from FTA leaf presses of diseased cassava leaves sampled in Western Kenya. The amplified PCR product was cloned into pGEM-T Easy vector (Promega), the DNA amplified by miniprep and digested with *Eco*RV for 1.5 hrs at 37°C. Unique bands generated by this restriction enzyme facilitate identification of single infections with EACMV and ACMV (lanes 1–3 and 5 respectively) and a plant co-infected with both geminivirus species (lane 4). M: marker, +C: positive control, W: water control.

Having demonstrated efficacy of FTA as a robust tool for sampling and recovery of high fidelity geminivirus DNA from greenhouse-grown material, the technology was tested in farmers' fields in East Africa. Leaves from cassava and maize plants symptomatic for cassava mosaic disease (CMD) and Maize streak virus (MSV) (Fig. 1a and 1b) were pressed onto FTA cards in Malawi and Western Kenya. Samples were returned to the DDPSC and processed as described above. Strong signals were recovered in all seven maize samples tested (Fig. 4a) using primers designed to amplify a 500 bp fragment from the conserved region of this monopartite geminivirus (Table 1). Likewise, all cassava samples collected in Malawi proved positive for the presence of EACMV-like geminivirus species (Fig. 4b). DNA eluted from FTA-pressed samples of symptomatic cassava leaves from Western Kenya was amplified using Universal primers UniF and UniR. The amplified 2.8 kb product was isolated from the agarose gel and cloned into pGEM-T Easy vector. Viral DNA was amplified by miniprep and subjected to restriction digestion with *EcoR*V. This enzyme is known to digest all ACMV-like and EACMV-like geminiviruses into unique polymorphic patterns, making it a useful tool for diagnostic analysis of CMD infections to the species and strain level [9]. Of the five plants analyzed in this manner, three were found to contain only EACMV-like viruses, one to be infected with ACMV and one to contain a dual infection with EACMV and ACMV (Fig. 4c).

### Use of FTA technology for sampling, retrieval and analysis of RNA viruses

**Figure 5:**
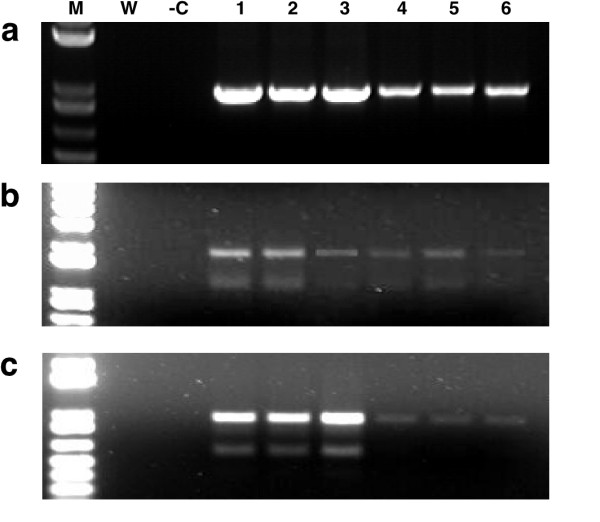
**RT-PCR amplification of three RNA viral pathogens recovered from diseased *N. benthamiana *leaves pressed on FTA cards**. Traditional isolation methods (lanes 1–3) were compared to RNA eluted from leaf pressed onto FTA cards (lanes 4–6) (a) a 900 bp fragment of tobacco mosaic virus (b) 1.5 & 2.1 kb fragments of tobacco etch virus (c) 1.5 & 2.1 kb fragments of potato virus Y. In all cases, although generating signals of lower strength compared to traditional RNA isolation methods, RNA eluted from FTA cards proved suitable for detection by RT-PCR analysis. M: marker -C: positive control, W: water control.

Since RNA viruses are responsible for the majority of viral diseases in plants, we investigated the efficacy of FTA technology for sampling and retrieval of the commonly studied virus Tobamovirus; Tobacco mosaic virus (TMV) and two potyviruses; Potato virus Y (PVY) and Tobacco etch virus (TEV) potyviruses that infect a large number of plant species. Leaves of *N. benthamiana *symptomatic for these pathogens were pressed onto FTA cards. PCR amplification of cDNA generated from viral RNA eluted from FTA cards was compared to that isolated via standard methods (where 100–200 mg of leaf tissue was used to isolate total RNA) [10,11]. In all cases PCR signals of the predicted sizes were obtained from both methods (Fig. 5). Signal strength generated from FTA derived samples was lower for all three viruses compared to that for RNA isolated by conventional methods. The lower signal strength from FTA samples reflected differences in the amount of RNA obtained by the two methods, and subsequently used as template for cDNA synthesis (40–60 ng/μl from FTA eluted samples compared to 0.4–1.0 μg/μl for conventional isolation). Nevertheless, signals obtained from the FTA cards were sharp and discrete, most especially for TMV (Fig. 5a), demonstrating that this technology is applicable as an efficient way of sampling, indexing, retrieving and detecting plant RNA viruses from infected plants.

### Use of FTA for the detection of integrated transgenes

**Figure 6:**
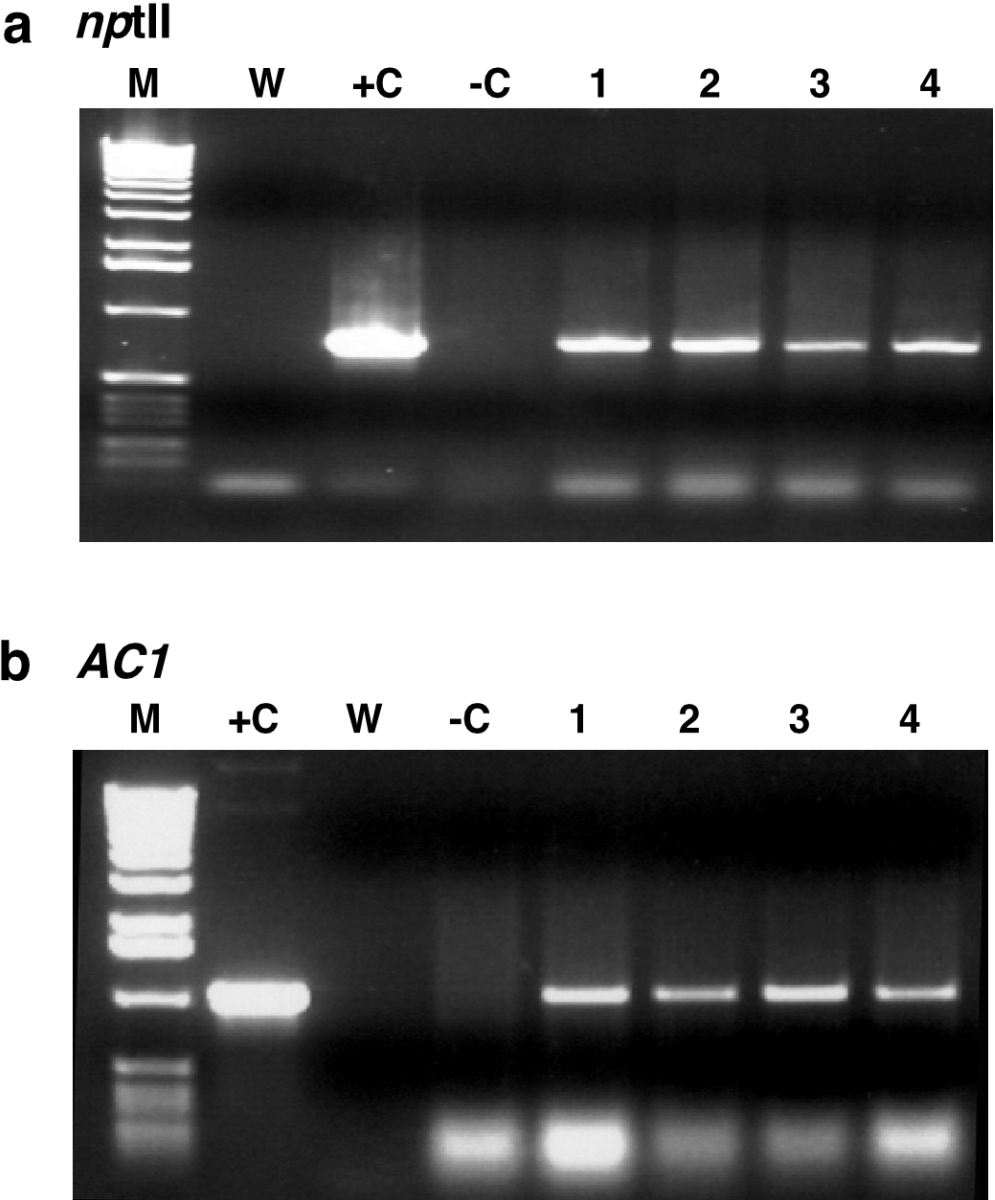
**PCR amplification of integrated transgene sequences from cassava**. (a) amplification of a 800 bp fragment of the *npt*II selectable marker gene from transgenic plants of cassava cv. 60444 using primers MP141 and MP142 (b) amplification of the 1070 bp *AC1 *transgene integrated into transgenic plants of cv. 60444 using primers AC1F and ACR (Chelleppan *et al., *2004) (Table 1).

Simple and cost effective tools for monitoring transgenic plants in the field is becoming increasingly important as more developing countries initiate field trials of genetically modified crops. A major goal of the DDPSC is to employ pathogen derived resistance strategies to engineer cassava for elevated resistance to cassava mosaic disease [12] and to test these by carrying out field trials in Africa. In such circumstances it is necessary to assess not only infection of such plants by geminiviruses but also to have simple methods to sample and confirm the transgenic nature of plants within the field. We thus assessed the suitability of FTA technology to act as a reliable tool for PCR amplification of integrated transgene sequences. Leaf tissues from transgenic, virus-free cassava plants were pressed onto FTA cards. Single, 2 mm diameter punches were removed and processed with TE buffer and FTA Purification Reagent, but not subjected to an elution step. Instead, the punch was included in the PCR reaction mix, in addition to primers designed to amplify the *AC1*and *nptII *transgenes (Table 1) [12]. Amplification signals were successfully generated for both transgenes in all plants tested (Fig. 6), indicating that FTA technology is suitable for sampling and detecting both geminivirus-derived and non-geminivirus-derived genomic nucleotide sequences directly from plant tissues.

## Conclusion

The above studies demonstrate that FTA technology is effective for sampling, storage and retrieval of viral pathogens from infected plant tissues growing under the greenhouse and field conditions. Storage and transport of purified nucleic acids on paper for subsequent elution has been common practice for many years. The important advantage brought by FTA technology is the ability to fix and reliably preserve nucleic acids within untreated host tissues. Benefits of this technology are realized at both the sampling and processing phases. Sampling plant material with FTA cards is reduced to simple, on-site hand pressing and is thus rapid and uncomplicated. The ability to store pressed and fixed samples at ambient temperatures also significantly reduces concerns regarding nucleic acid degradation during sampling and storage. Combined with the lack of bulk offered by paper-based collection, the potential number of samples that can be collected within a given time and location is significantly increased compared to conventional methods.

Effective retrieval from FTA cards of RNA and DNA viral sequences, plus that of plant genomic DNA, has been demonstrated here. Such capacity eliminates the need for traditional multi-step extraction and purification procedures (some of which require the use of hazardous chemicals) and the requirement for refrigeration and centrifugation equipment. Processing of plant samples is reduced instead to a simple series of washes within a single Eppendorf tube. Importantly, all downstream analytical procedures remain unchanged from existing systems, meaning that no new investment in protocol development is associated with the application of FTA. The technology is also economically effective with samples costing less than $0.75 each reach the nucleic acid elution stage. Nucleic acid elution and subsequent analysis requires removal of only a few punches from the tissue press, allowing the remainder to be archived for future reference. The length of time that plant viral genomes can be stored on FTA cards was not tested within this study, but research by the manufacturers provides evidence that under ambient conditions the integrity of DNA within pressed tissue samples is maintained for more than fourteen years [1]. If vacuum packed and placed within a fire-proof cabinet, FTA cards provide a long term, low cost, low risk archiving system for viral pathogen and plant genomic samples.

The benefits described above have important implications for improving the efficiency of sampling plant tissues in the laboratory environment but increase greatly when working in the field, and most especially within remote areas and in developing countries where access to laboratory facilities, chemicals and equipment is limiting. Results obtained from FTA sampled material were effective and reproducible in our hands from the four plant species studies, whether collected from the greenhouse or returned to the USA from farmer's fields in Africa. Predicted PCR products were obtained in 100% and 80% of the cassava leaf samples collected from the greenhouse and field respectively, with all the MSV-infected field grown maize plants sampled yielding viral sequences. In all cases, FTA cards yielded viral nucleic acids of a quality equivalent to that obtained from tradition chemical extraction methods. A full range of diagnostic tools could be applied to viruses eluted from FTA cards, including PCR, RT-PCR, restriction analysis, cloning and nucleotide sequencing. The studies described here demonstrate that FTA offers a simple, sensitive and specific tool appropriate for the diagnosis and molecular characterization of plant viral pathogens isolated from plant tissues and transgene sequences integrated into the plant genome. We conclude that the application of this technology has the potential to significantly increase ability to bring modern analytical techniques to bear on the viral pathogens infecting crop plants.

## Methods

### Sampling symptomatic leaves with FTA cards

Young, symptomatic leaves were removed from infected plants and placed on FTA^® ^Classic Cards. A 16 cm^2 ^piece of parafilm was placed over the plant tissue and the rounded end of a plastic test tube used to apply moderate downward pressure with a slight twisting action until sap penetrated the reverse side of the FTA paper (Fig. 1c). Cards were dried at room temperature overnight and stored in paper bags until required for processing.

### Elution and amplification of DNA viruses from FTA cards

Young leaves of cassava, maize and tomato infected with geminivuruses were pressed onto FTA cards as described above. Three 2 mm diameter punches were removed from each chlorophyll-stained region using a Harris 2.0 mm punch (Fig. 1d) and placed in a sterile 1.5 ml Eppendorf tube. Paper discs were washed in 300 μl of TE buffer for five minutes followed by sequential five-minute washes with 300 μl of 70% ethanol and FTA^® ^Purification Reagent. Punches were then transferred to a fresh 1.5 ml Eppendorf tube and allowed to dry for two hours at room temperature. Viral DNA was eluted by soaking the punches in 10–12 μl of elution buffer (10 mM Tris, 0.1 M EDTA, pH 8.5) for 20–30 minutes and stored at -20°C until required. Two microlitre aliquots of elute were used as template for PCR amplification in a 50 μl reaction volume. Primer sequences and PCR reaction conditions for the respective primers employed are provided in Table 1. DNA was also extracted by traditional methods based on those of Dellaporta et al. [1].

For serial dilutions, recombinant plasmid DNA carrying the B component of EACMCV [8] in quantities; 0.8, 0.4, 0.2, 0.16, 0.08, 0.05, 0.04, and 0.001 μg was mixed with 8 μl of sap extracted with distilled water from healthy cassava leaves, loaded onto FTA cards and allowed to dry at room temperature for two hours. The stained region from each sample was cut from the FTA paper and processed as described above to elute viral the DNA.

### Cloning and nucleotide sequencing of viral DNA eluted from FTA cards

Template DNA eluted from FTA cards and obtained from Dellaporta-based conventional extraction methods [1] was amplified by PCR as described above. PCR products were purified and cloned into the pGEM-T Easy vector (Promega) and sequenced in both orientations. DNA nucleotide sequences were compared by multiple alignment using Mega Align option of DNASTAR computer package. A corresponding fragment sequence of EACMCV DNA B from (GeneBank: (AF112355) was used as a reference.

### Elution and RT-PCR amplification of RNA viruses from FTA cards

Symptomatic leaves from tobacco plants infected with TMV, PVY and TEV were pressed onto FTA cards as described above. Eight disc, 2 mm in diameter were removed from the chlorophyll-stained region of each pressed sample and placed into a RNase-free/ DNase-free 1.5 ml Eppendorf tube. Five hundred μl of fresh RNA processing buffer (10 mM Tris-HCl, pH 8.0, 0.1 mM EDTA, 800 U/mL RNase Out™ {Invitrogen Life Technologies, Inc., USA} 200–250 μg/mL glycogen and 2 mM DTT) was added to each Eppendorf tube and incubated on ice with mixing every 5 min for a total of 30 mins. The paper discs were removed and eluted RNA precipitated using 1/10th volume of 3 M sodium acetate (pH 5.2), an equal volume of ice cold 100% isopropanol and incubated at -70°C for 30 mins. RNA was pelleted and washed with ice-cold 75% ethanol, dried and resuspended in 30 μl of DEPC-treated TE/ H_2_O. Isolation of RNA also performed by the above method directly from 100–200 mg of symptomatic fresh leaf tissue fresh.

For TMV, total RNA was used directly for cDNA synthesis, but for PVY and TEV that possess polyA tails, messenger RNA was purified from total RNA using Oligotex-dT (Qiagen) according to the manufacturer's instructions. cDNA was synthesized in a 20 μl reaction using superscriptIII reversetranscriptase (Invitrogen). For TMV, 0.4–1.0 μg total RNA was used per reaction with TMV specific TMV-R reverse primer (Table 1), and for PVY and TEV 30–100 ng mRNA was used with the potyvirus universal (POT1) reverse primer (3'-ACCACRTADCTBTTAcctag gtcag 5'). Subsequently, PCR was done on cDNA to amplify *CP *and *MP *sequences for TMV and the conserved region of *CP *and *NiB *from PVY and TEV potyviruses using specific primers (Table 1).

### PCR amplification of genomic DNA sequences from FTA cards

Leaf tissues from transgenic, virus-free cassava plants were pressed onto FTA cards as described above. Single, 2 mm diameter punches were removed from chlorophyll-stained regions and placed in a sterile 1.5 ml Eppendorf tube. Punches were washed sequentially for five minutes each with 300 μl of TE buffer, 70% ethanol and FTA Purification Reagent, followed by transfer to a fresh 1.5 ml Eppendorf tube where they were allowed to dry for two hours. Single punches were added to 50 μl PCR reaction mixes containing primers MP141 and 142 for the amplification of the *npt*II coding sequence, and primers AC1F and AC1R to amplify the *AC1 *transgene sequence [12] (Table 1).

## Competing interests

The authors declare that have no competing interests. Whatman International Inc. provided free samples of their FTA technology to facilitate early stages of the work reported here. None of the authors received payment from Whatman to undertake this research.

## Authors' contributions

JN developed FTA technology for isolation and detection of geminiviruses, carried out the cloning, sequencing and restriction analysis and produced the initial manuscript draft. NJT conceived the use to FTA for detection of DNA viruses, applied FTA for sampling cassava in the field in Africa, generated data on maize streak virus and use of FTA for PCR amplification of genomic sequences and prepared the final versions of the manuscript. JY carried out all RNA work described above. HA adapted FTA technology for detection of TYLCV and make discoveries to improve recovery of geminiviruses from FTA cards. JL conceived and contributed to the use of FTA in the field in Africa and provided critical input in drafting the manuscript. TA interpreted data, corrected the manuscript and provided supervision of JN. GT guided experimental design and corrected the manuscript. CMF applied FTA technology in the field to collect MSV from diseased maize plants, guided experimental design, conceived the use of FTA for detection of RNA viruses, provided overall supervision and financial support and corrected the manuscript.

## References

[B1] Dellaporta SL, Wood J, Hicks JB (1983). A plant DNA minipreparation: version II. Plant Mol Biol Rep.

[B2] Whatman (2004). Application of FTA-based technology for sample collection, transport, purification and storage of PCR-ready plant DNA. http://www.whatman.co.uk/repository/documents/s3/usFtaPlantDna.pdf.

[B3] Zhong KJY, Salas CJ, Shafer R, Gubanov A, Gasser RJR, Magill AJ, Forney JR, Kain KC (2001). Comparison of IsoCode STIX and FTA gene guard collection matrices as whole- blood storage and processing devices for diagnosis of malaria by PCR. J Clin Microbiol.

[B4] Smith LM, Burgoyne LA (2004). Collection, archiving and processing DNA from wildlife samples using FTA^® ^databasing paper. BMC Ecology.

[B5] Tsukaya H (2004). Gene flow between *Impatiens radicans *and *I. javensis*(Balsaminaceae) in Gunung Pangrango. Am J Bot.

[B6] Drescher A, Graner A (2002). PCR-genotyping of barley seedlings using DNA samples from tissue prints. Plant Breed.

[B7] Pita JS, Fondong VN, Sangaré A, Kokora RNN, Fauquet CM (2001). Genomic and biological diversity of the African cassava geminiviruses. Euphytica.

[B8] Fondong V, Pita JS, Rey MEC, de Kochko A, Beachy RN, Fauquet CM (2000). Evidence of synergism between African cassava mosaic virus and the new double recombinant geminivirus infecting cassava in Cameroon. J Gen Virol.

[B9] Legg JP, Fauquet CM (2004). Cassava mosaic geminiviruses in Africa. Plant Mol Biol.

[B10] Souto ER, Sim J, Chen J, Valverde RA, Clark CA (2003). Properties of strains of sweet potato feathery mottle virus and two newly recognized potyviruses infecting sweet potato in United States. Plant Dis.

[B11] Colinet D, Colinet M, Nguyen J, Kummert P, Lepoivre A, Feng ZX (2003). Differentiation among potyiruses infecting sweet potato based on genus-and virus-specific transcription polymerase chain reaction. Plant Dis.

[B12] Chellappan P, Masona MV, Vanitharani R, Taylor NJ, Fauquet CM (2004). Broad Spectrum Resistance to ssDNA viruses associated with transgene-induced gene silencing in cassava. Plant Mol Biol.

